# Colibactin-producing *Escherichia coli* enhance resistance to chemotherapeutic drugs by promoting epithelial to mesenchymal transition and cancer stem cell emergence

**DOI:** 10.1080/19490976.2024.2310215

**Published:** 2024-02-19

**Authors:** Guillaume Dalmasso, Antony Cougnoux, Tiphanie Faïs, Virginie Bonnin, Benoit Mottet-Auselo, Hang TT Nguyen, Pierre Sauvanet, Nicolas Barnich, Marine Jary, Denis Pezet, Julien Delmas, Richard Bonnet

**Affiliations:** a Inserm U1071, USC-INRAe INRAE USC 1382, Microbes, Intestin, Inflammation et Susceptibilité de l’Hôte (M2iSH), Centre de Recherche en Nutrition Humaine Auvergne, Université Clermont Auvergne, Clermont-Ferrand, France; b Laboratoire de Bactériologie, Centre Hospitalier Universitaire, Clermont-Ferrand, France; c Centre de référence de la résistance aux antibiotiques, Centre Hospitalier Universitaire, Clermont-Ferrand, France; d Service de Chirurgie Digestive, Centre Hospitalier Universitaire, Clermont-Ferrand, France

**Keywords:** Colorectal cancer, colibactin, *pks*, chemotherapy resistance, cancer stem cell, microbiota, *Escherichia coli*, CoPEC

## Abstract

Human colorectal cancers (CRCs) are readily colonized by colibactin-producing *E. coli* (CoPEC). CoPEC induces DNA double-strand breaks, DNA mutations, genomic instability, and cellular senescence. Infected cells produce a senescence-associated secretory phenotype (SASP), which is involved in the increase in tumorigenesis observed in CRC mouse models infected with CoPEC. This study investigated whether CoPEC, and the SASP derived from CoPEC-infected cells, impacted chemotherapeutic resistance. Human intestinal epithelial cells were infected with the CoPEC clinical 11G5 strain or with its isogenic mutant, which is unable to produce colibactin. Chemotherapeutic resistance was assessed *in vitro* and in a xenograft mouse model. Expressions of cancer stem cell (CSC) markers in infected cells were investigated. Data were validated using a CRC mouse model and human clinical samples. Both 11G5-infected cells, and uninfected cells incubated with the SASP produced by 11G5-infected cells exhibited an increased resistance to chemotherapeutic drugs *in vitro* and *in vivo*. This finding correlated with the induction of the epithelial to mesenchymal transition (EMT), which led to the emergence of cells exhibiting CSC features. They grew on ultra-low attachment plates, formed colonies in soft agar, and overexpressed several CSC markers (e.g. CD133, OCT-3/4, and NANOG). In agreement with these results, murine and human CRC biopsies colonized with CoPEC exhibited higher expression levels of OCT-3/4 and NANOG than biopsies devoid of CoPEC. **Conclusion**: CoPEC might aggravate CRCs by inducing the emergence of cancer stem cells that are highly resistant to chemotherapy.

## Introduction

In recent years, significant advances have been reported in the treatment of many tumor types, including colorectal cancer (CRC).^1^ Despite substantial progress, resistance to therapy remains a major challenge and the leading cause of treatment failure, due to cancer cells’ resistance to drug treatment resulting in tumor recurrence and metastasis. Cancer stem cells (CSCs) – subpopulations of cancer cells thought to drive tumor growth – are a major cause of cancer therapy failure.^2,3^ CSCs share similar characteristics with normal stem cells, such as quiescence, self-renewal ability, and multi-lineage differentiation, which result from a balance between the quiescence, symmetric division, and asymmetric division of CSCs.^2,3^

Causing a loss of the epithelial cell phenotype and a gain of mobility,^4–6^ epithelial to mesenchymal transition (EMT) has been initially investigated for its enhancing effect on cell migration. EMT is also thought now to be involved in carcinoma cells’ acquisition of stem-like properties^7–10^ and resistance to anti-cancer drugs.^11–15^ As a transient and reversible cellular process, EMT could allow interconversions of CSCs and non-CSCs, by decreasing (e.g., E-cadherin) and increasing the expressions of EMT factors (e.g., vimentin, N-cadherin, ZEB, SNAIL, and TWIST).^2,16^ Among the EMT factors, transcription factors (e.g., TWIST, ZEB, and SNAIL) are particularly important for EMT initiation,^17^ stemness acquisition,^7,9,10,16^ and resistance to chemotherapy.^18^

Recent experimental studies regarding colorectal tumors have revealed the impact of microbiota composition on optimal responses to cancer therapies^19–22^ and immunotherapy.^23,24^ The identification of key microbiota members responsible for anti-cancer drug resistance has only been sparsely investigated, with the exception of *Fusobacterium nucleatum*, which mediates CRC chemoresistance against small drug chemotherapeutics via the autophagy pathway.^25–29^

*Escherichia coli*, a versatile subdominant member of the gut microbiota responsible for intestinal and extra-intestinal infections,^30,31^ is frequently isolated in biopsies of colorectal tumors,^32–35^ suggesting interactions between CRC and *E. coli*. Importantly, at least 50% of colorectal cancer (CRC) biopsies are colonized by *E. coli* strains harboring the *pks* genomic island,^34,36,37^ which is responsible for the synthesis of the toxin colibactin. Colibactin-producing *E. coli* (CoPEC) increases tumors in CRC mouse models^36,38–40^ and induces DNA interstrand crosslinks,^41^ DNA double-strand breaks,^36,42^ genomic instability,^43^ cell cycle arrest,^42^ and cellular senescence.^38,39^ Senescent cells produce a senescence-associated secretory phenotype (SASP) that, in turn, produces proinflammatory and growth factors, which are involved in the growth of tumor xenografts in mice transiently infected with CoPEC. Furthermore, senescence as well as SASP stigmas have been observed in human CRC biopsies colonized by CoPEC.^38^ In addition, a recent study showed that CoPEC induces a specific DNA mutation pattern that can be found in human CRC biopsies, demonstrating for the first time that bacteria could play a driving role in CRC pathogenesis.^44^

In this work, we investigated the fate of human colon cancer cells after infection with CoPEC and their consequent susceptibility to anticancer chemotherapy. We found that CoPEC promoted chemoresistance in colon cancer cells by promoting stemness and EMT features.

## Results

### Tumors colonized by CoPEC exhibited high expression levels of NANOG and OCT-3/4

**Figure 1.**
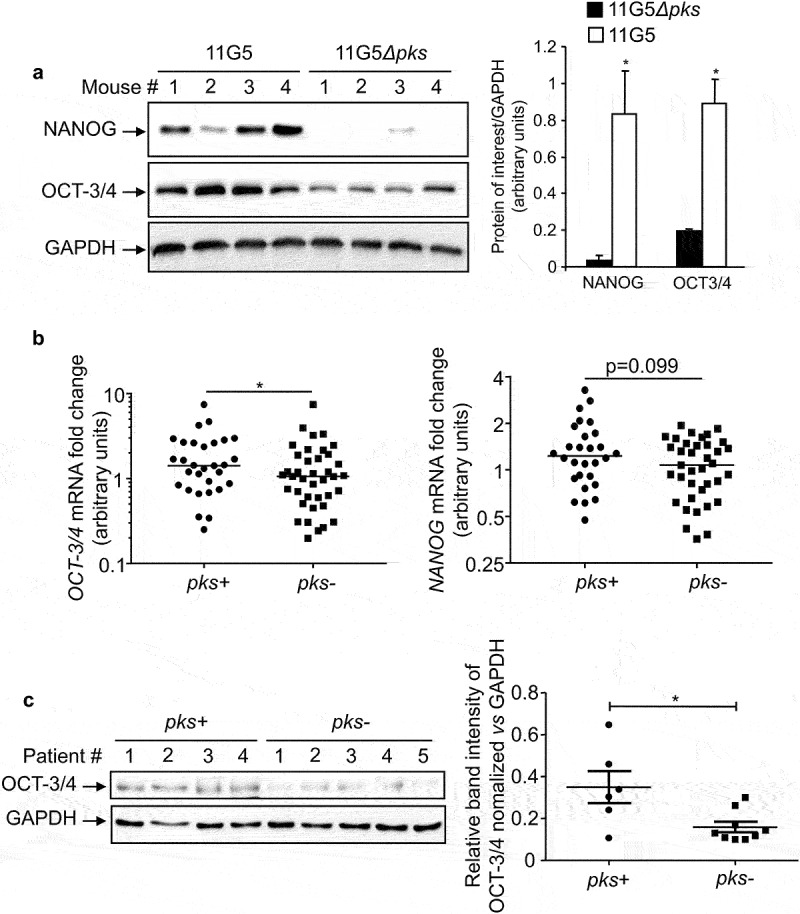
*In vivo* overexpression of CSC markers in response to CoPEC infection. (a) Western blot analysis of NANOG and OCT-3/4 expression in tumors collected from AOM/DDS-treated mice infected with the 11G5 strain or the 11G5*Δpks* strain. Bar graphs on the right represent quantification of bands density using ImageJ software. Values represent means ± SEM; *p < .05. (b) *OCT-3/4* and *NANOG* mRNA levels in the human non-tumoral colonic mucosa colonized by CoPEC (*pks+*) (*N* = 30), or by *E. coli* that did not carry the *pks* island (*pks-*) (*N* = 39), were quantified using qRT-PCR. Medians are represented on the graph; *p < .05. (c) Expression of OCT-3/4 in colonic tumors colonized by CoPEC (*pks+*) (*N* = 6), or by *E. coli* that did not carry the *pks* island (*pks-*) (*N* = 9), was analysed using Western blot. The graph represents the quantification of band intensity using the Image Lab Software from Bio-Rad; *p < .05.

The intracellular proteins NANOG (nanog homeobox) and OCT-3/4 (octamer-binding transcription factor) are key stem cell and prognostic markers.^45–47^ We therefore investigated the expression of these markers in colonic tumors collected from the AOM/DSS CRC mouse model and from human biopsies colonized by *E. coli*. The AOM/DSS-treated mice were colonized by clinical colibactin-producing *E. coli* (CoPEC) reference strain 11G5 or its isogenic mutant 11G5*Δpks*, which is unable to produce colibactin (the *clbQ* gene of the *pks* island has been deleted).^38^ Expressions of NANOG and OCT-3/4 were greatly increased in colonic tumors collected from mice colonized by 11G5 compared to mice harboring colonic tumor colonized by 11G5*Δpks* (Figure 1a). Similar investigations were performed using human colonic mucosa and human colonic tumors, which did not significantly differ in their tumor node metastasis stage, neoplastic grade, inflammatory score, or quantity of associated *E. coli*. ^39^ We found that colonic mucosa isolated from patients colonized with CoPEC harbored higher mRNA levels of *OCT-3/4* and *NANOG* compared to those colonized by *E. coli* devoid of *pks* (Figure 1b). Protein expression levels of OCT-3/4 were also significantly increased in tumors colonized by CoPEC (Figure 1c). However, the NANOG protein was undetectable by Western blot (data not shown). These results suggested that CoPEC fosters the emergence of CSCs *in vivo* and therefore might enhance chemoresistance.

### CoPEC promoted resistance to anti-cancer chemotherapy in vitro and in vivo

**Figure 2.**
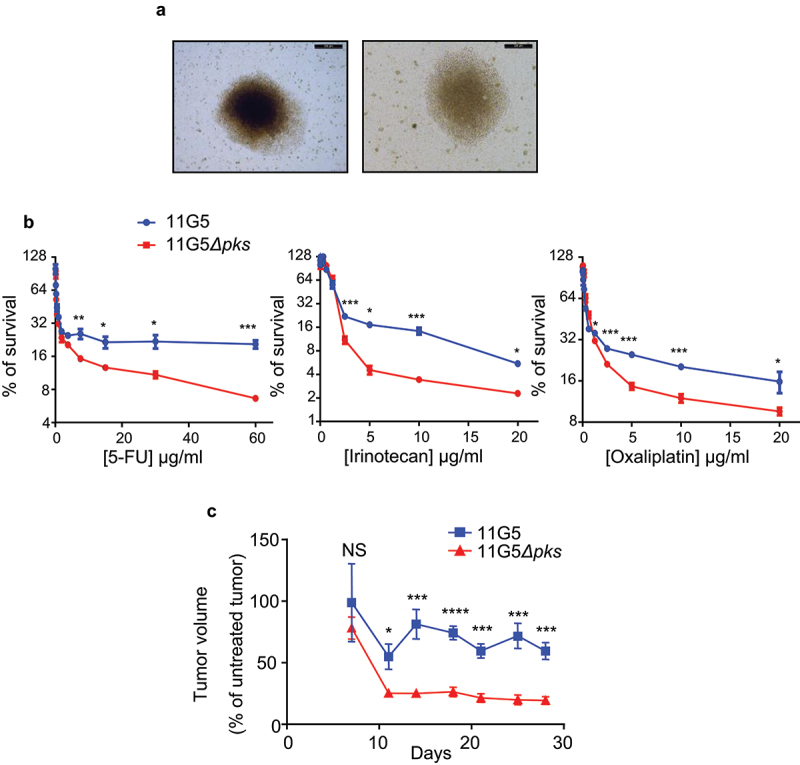
Human colon cancer cells made senescent by 11G5 growth at 3 weeks post-infection and are more resistant to chemotherapeutic drugs both *in vitro* and in a xenograft mouse model. (a-c) HT-29 cells were infected with the 11G5 strain or the 11G5*Δpks* strain, and 3-week post-infection cells were used. (a) Representative pictures of 11G5-infected cells at 3 weeks post-infection. (b) Cells were trypsinized, seeded on 96-well plates, and exposed to various doses of chemotherapeutic drugs for 1 week. Cellular viability was assessed by MTT assay. Untreated cells were used to represent 100% viability. Data are means ± SEM of eight replicates and are representative of three independent experiments. *p < .05; **p < .005; ***p < .001. (c) Three weeks post-infection, 10^6^ cells were subcutaneously injected into the dorsal flaps of 5-week-old nude mice. Seven days post-engraftment, mice received 30 mg/kg of 5-FU twice weekly for 3 weeks. Tumor sizes were measured using a caliper twice weekly. *N* = 6 mice/group. Data are means ± SEM. NS, not significant; *p < .05; ***p < .001; ****p < .0001.

Human colon carcinoma HT-29 cells were infected *in vitro* with the 11G5 strain or the isogenic mutant 11G5*Δpks*.^38^ Five days post-infection, all the cells infected with 11G5, unlike the cells infected with 11G5*Δpks*, exhibited megalocytosis – an expected morphological change relating to the cellular senescence induced by CoPEC—, as previously published.^38^ The senescent cells were maintained in culture, and their morphology was monitored using a transmitted light microscope twice weekly. Between 2- and 3-weeks post-infection, we recurrently observed a rebound in the growth of 11G5-infected cells, which formed 3D spheroid cellular clusters (Figure 2a), while 11G5*Δpks*-infected cells, like uninfected cells, grew as monolayers requiring frequent trypsinization. The susceptibility of infected cells to chemotherapeutic drugs was next assessed at 3 weeks post-infection. As shown in Figure 2b, 115G5-infected cells were more resistant to the first- (5-fluorouracil or 5-FU) and second-generation (irinotecan and oxaliplatin) chemotherapy used for CRC treatment compared to 11G5*Δpks*-infected cells. To validate the results obtained *in vitro*, the susceptibility of CoPEC-infected cells to anti-cancer chemotherapy was analyzed in a xenograft mouse model. Three weeks post-infection, cells were engrafted into nude mice, and the mice received administration of 5-FU as described in Materials and Methods. According to the results obtained *in vitro*, tumors derived from 11G5-infected cells were significantly more resistant to 5-FU than those from 11G5*Δpks*-infected cells (Figure 2c).

Overall, the results showed that cancer cells infected by CoPEC, despite the senescence induced by the genotoxic bacteria, form 3D spheroids and become chemoresistant.

### CoPEC activated EMT and induced the emergence of CSCs

**Figure 3.**
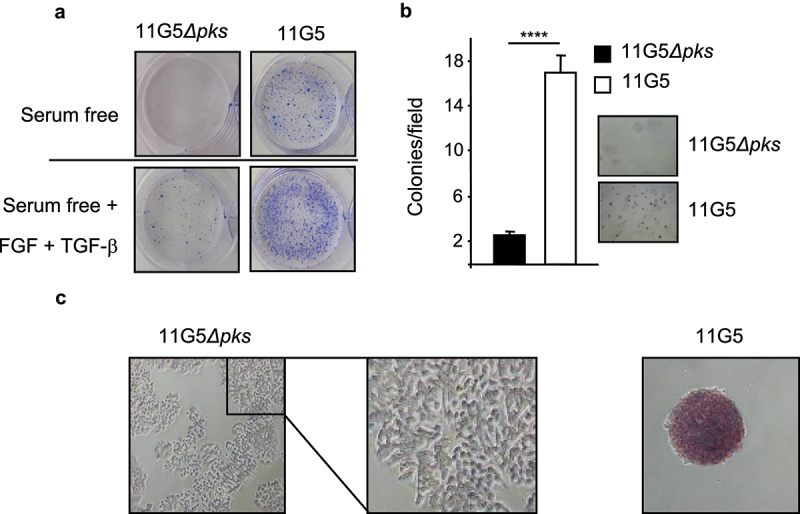
Human colon cancer cells made senescent by 11G5 exhibited rebound growth and features of CSCs at 3 weeks post-infection. (a–c) HT-29 cells were infected with the 11G5 strain or the 11G5*Δpks* strain, and 3-week post-infection cells were used. (a) Cells were trypsinized and seeded on ultra-low attachment plates in the presence or absence of EGF and FGF. Representative pictures are shown. (b) Cells were trypsinized and seeded in a culture medium with 0.7% soft agar. After 2 weeks, colonies were counted (bar graph). Representative pictures are shown to the right of the bar graph. Values represent means ± SEM; ****p < .0001. (c) Cells were incubated with a substrate of alkaline phosphatase. A brown color signaled substrate degradation by the alkaline phosphatase. Representative pictures are shown.

Since chemoresistance and 3D spheroid formation are features of CSCs,^2,48,49^ we investigated stem cell markers in human colon cancer cells that were made resistant to chemotherapy by CoPEC infection, as described above. Compared to 11G5*Δpks*-infected cells, CoPEC-infected cells displayed enhanced growth on ultra-low attachment plates (Figure 3a) and an ability to form colonies in soft agar (Figure 3b), which are hallmarks of CSCs.^50^ In addition, 11G5 induced an increase in alkaline phosphatase activity – a well-known stem cell marker^51,52^ (Figure 3c).

**Figure 4.**
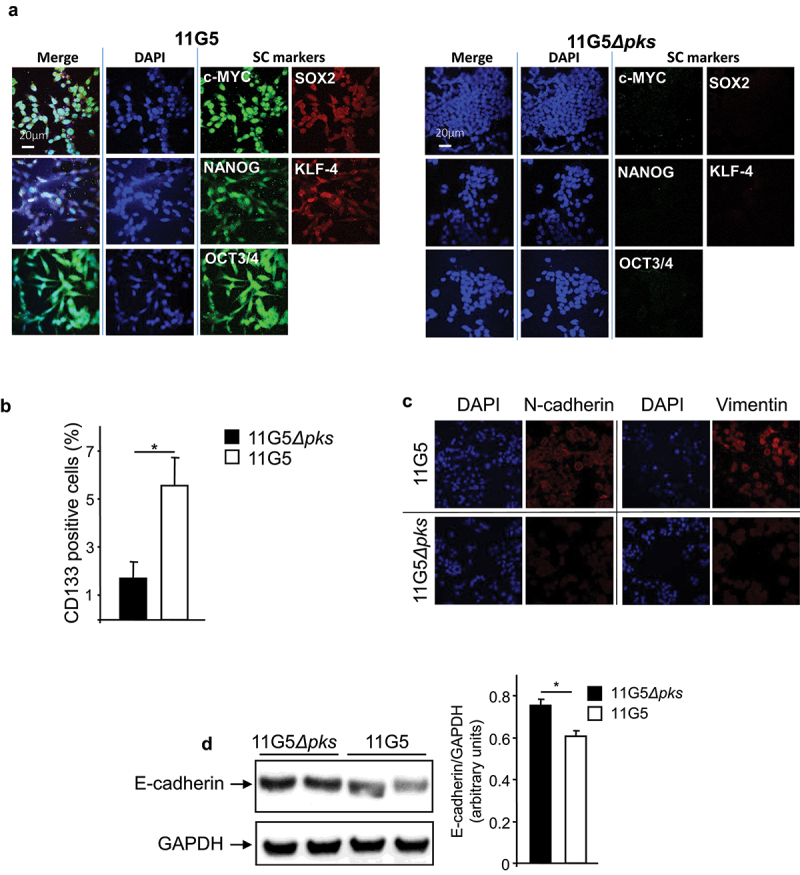
11G5 infection of human colon cancer cells fostered the emergence of cells expressing CSC and EMT markers at 3 weeks post-infection. (a–d) cells were infected with the 11G5 strain or the 11G5*Δpks* strain, and 3-week post-infection cells were used. (a) Spheroids resulting from 11G5 infection were mechanically disrupted and then stained for the transcription factors involved in cellular reprogramming by immunofluorescence [red (SOX-2 and KLF-4) or green (c-MYC, NANOG, OCT3/4)]. Nuclei were stained with DAPI (blue). (b) Quantification of cells expressing CD133. Values represent means ± SEM; *p < .05. (c) Immunofluorescent labelling of the EMT markers N-cadherin or vimentin (red). Nuclei were stained with DAPI (blue). (d) The EMT marker E-cadherin was analysed using Western blot. Bar graph on the right represents quantification of bands density using ImageJ software. Values represent means ± SEM; *p < .05.

To confirm the emergence of CSCs from cultures of CoPEC-infected cancer cells, we investigated the expression and sub-cellular location of NANOG, OCT-3/4, SOX2 (SRY-box transcription factor 2), KLF-4 (Kruppel-like factor 4), and c-MYC, which are key transcription factors in cell reprogramming and stem cell pluripotency^52^ and used to identify CSCs in a variety of cancers, including CRC.^53^ Immunofluorescence experiments revealed the expression and accumulation of these four transcription factors in the nuclei of cells at 3 weeks post-infection with 11G5 (Figure 4a left panel). Their expression was almost undetectable in 11G5*Δpks*-infected cells (Figure 4a right panel). Furthermore, CD133, a classic marker of colon CSCs, was also found to be overexpressed in 11G5-infected cells (Figure 4b). Since EMT is a cellular dedifferentiation process tightly linked to CSC formation and drug resistance,^2,54^ we investigated the expression of EMT markers in human colon cancer cells at 3 weeks post-infection. As shown in Figures 4c, d, 11G5 infection, unlike 11G5*Δpks* infection, induced an increase of both N-cadherin and vimentin expression, and a decrease of E-cadherin expression – a pattern usually observed during EMT.^6^

In a previous study, transcomplementation of *clbQ* gene in the 11G5*ΔclbQ* strain, restored the wild-type phenotype (e.g. cellular megalocytosis, cellular senescence, etc.).^38^ Here, we also double-checked that the observed phenotypes/markers upon 11G5*ΔclbQ* infection were due to the absence of a functional *pks* island. For that, HT-29 cells were infected with a transcomplementated 11G5*ΔclbQ* strain (11G5*ΔclbQ:clbQ*). As shown in Supplemental Figure 1, the 11G5*ΔclbQ:clbQ* strain induced the same cellular consequences as the wild-type 11G5 strain (a decrease in E-cadherin expression, an increase in NANOG expression, and an increase in chemotherapy drugs resistance).

Overall, our results suggested that the post-senescence cancer cell growth observed after CoPEC infection was associated with both EMT induction and CSC emergence.

### CoPEC-associated SASP induced EMT and the emergence of CSCs

**Figure 5.**
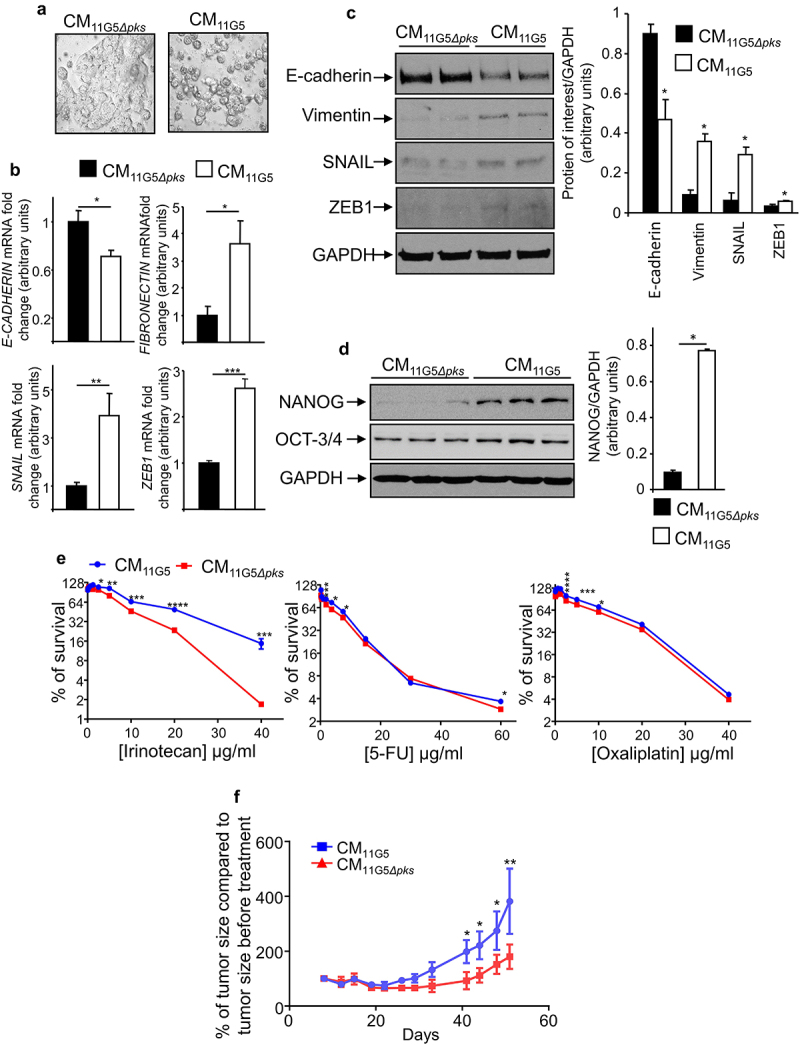
The SASP induced by 11G5 infection promoted the expression of CSC and EMT markers leading to chemoresistance in non-infected human colon cancer cells. (a–f) Cells were infected with the 11G5 strain or the 11G5*Δpks* strain. Five days post-infection, conditioned media (CM) derived from infected cells were collected and used to culture uninfected cells for 3 days (panel B), 5 days (panels A, C, D, F), 7 days (panel E). CM_11G5_, CM derived from 11G5-infected cells; CM_11G5*Δpks*_, CM derived from 11G5*Δpks*-infected cells. (a) Representative picture of cells incubated with the indicated CM. (b) *E-cadherin*, *fibronectin*, *SNAIL*, and *ZEB1* mRNA levels were quantified using qRT-PCR. Values represent means ± SEM. *p < .05; **p < .01; ***p < 0.001. (c) EMT and (d) the NANOG CSC markers were analysed using Western blot. Bar graphs on the right represent quantification of bands density using ImageJ software. Values represent means ± SEM; *p < .05. (e) Uninfected cells were cultured for 1 week with the indicated CM supplemented with various concentrations of chemotherapeutic drugs. Cell viability was assessed using MTT assay. Untreated cells were used to represent 100% viability. Values represent means ± SEM. *p < .05; **p < .01; ***p < .001; ***p < .0001. (f) After 5 days of culture in the presence of the indicated CM, 10^6^ HT-29 cells were subcutaneously injected into the dorsal flaps of 5-week-old nude mice. 7 days post-engraftment, mice received 30 mg/kg of irinotecan twice weekly for 3 weeks. Tumor sizes were measured using a caliper twice weekly. *N* = 6 mice for CM_11G5_ group and *N* = 5 mice for CM_11G5*Δpks*_ group. Data are means ± SEM. *p < .05; **p < .01.

To determine the mechanisms sustaining CSC emergence in response to infection with CoPEC, we analyzed the role of the SASP produced by CoPEC-infected cells. A cell culture medium conditioned by 11G5-infected cells was collected at 5 days post-infection, the delay being necessary to obtain senescent cells in response to CoPEC infection as previously described.^38^ The resulting conditioned media (CM), which contained the cellular SASP induced by CoPEC, were incubated with non-infected human colon cancer cells. CM derived from 11G5-infected cells (CM_11G5_), unlike CM derived from 11G5*Δpks*-infected cells (CM_11G5*Δpks*_), induced a change in the morphology of the cells. Instead of growing as a regular monolayer, the cells were rounder and harbored decreased intercellular contacts (Figure 5a). Compared to CM_11G5*Δpks*_ stimulation, CM_11G5_ induced a significant decrease in *E-cadherin* mRNA level, as well as a significant increase in *SNAIL*, *ZEB1*, and *fibronectin* mRNA levels (Figure 5b). A tendency of increasing *vimentin* and *N-cadherin* mRNA levels was observed upon CM_11G5_ *versus* CM_11G5_*_Δpks_* stimulation (supplemental Figure 2). Furthermore, CM_11G5_ also induced a decreased expression of the protein E-cadherin and an increased accumulation of the proteins vimentin, SNAIL, and ZEB1 (Figure 5c). These indicated that EMT was induced by CM_11G5_. The NANOG stem cell marker, which is a key regulator of cellular reprogramming fostering CSC traits^55,56^ observed in this work in mouse and human CoPEC-infected CRC tumors, was also overexpressed in response to CM_11G5_ compared to CM_11G5*Δpks*_ (Figure 5d). Finally, we investigated the impact of CM on cells’ resistance to chemotherapeutic drugs. As shown in Figure 5e, CM_11G5_ significantly increased *in vitro* cellular resistance to irinotecan, and tended to increase resistance to 5-FU and to oxaliplatin, compared to CM_11G5*Δpks*_. Similarly, mouse xenografts from human colon cancer cells, treated over 5 days before engraftment with CM_11G5_, were more resistant to irinotecan than those from cells treated over 5 days with CM_11G5*Δpks*_ (Figure 5f).

As expected, CM-derived from HT-29 cells infected with 11G5*ΔclbQ:clbQ* induced, like CM_11G5_, a decrease in E-cadherin, an increase in NANOG, and an increased resistance to irinotecan (Supplemental Figure S3).

Our results suggested an active role of senescence and SASP in the induction of EMT, the emergence of CSCs and drug resistance in response to CoPEC infection.

## Discussion

The majority of patients with advanced CRC are initially responsive to the combined chemotherapy including 5FU, irinotecan, and oxaliplatin, which constitute first-line therapy for CRC. However, the recurrence of chemoresistant disease following therapy remains an intractable problem and represents a major impediment to reducing the morbidity and mortality attributable to malignant tumors. In addition, colon cancer patients are generally not responsive to novel immune checkpoint therapy.^57,58^ It is therefore of paramount importance to elucidate the mechanism of chemotherapy resistance in CRC patients. Bacteria overrepresented in human CRC biopsies, such as *E. coli*, can modulate the physiology of cancer cells. However, our understanding of the CRC and microbe relationships and their influences on therapeutic outcome is in its infancy. The present study has uncovered a novel role for CRC-associated *E. coli* in modulating therapeutic efficacy.

To address this issue, investigators have traditionally focused on elucidating the cell-intrinsic mechanisms that render tumors refractory to chemotherapeutics. However, cancer resident in organs throughout the body do not develop in isolation. Tumor cells arise in the context of nonmalignant cellular and non-cellular components of a tissue, defined as the tumor microenvironement (TME). The importance of TME in cancer initiation and progression is well established, and TME is now a key target for treatments.^59–61^ In addition, tumors that arise at epithelial barrier surfaces of the body such as CRC harbor an extensive microbiota in the TME, and the importance of these microbes in cancer is now widely appreciated.^62–66^ Bacteria overrepresented in human CRC biopsies, such as *Bacteroides fragilis*, *Fusobacterium nucleatum*, or *E. coli*, can modulate the physiology of cancer cells.^67–70^ However, our understanding of the TME and microbe relationships and their influence on therapeutic outcomes is in its infancy. The present study has uncovered a novel role for CRC TME-associated *E. coli* in modulating therapeutic efficacy.

CoPEC, unlike *E. coli* devoid of *pks* and consequently unable to produce colibactin, promoted (*in vitro* and *in vivo*) multi-drug resistance, upregulation of EMT markers, and the emergence of cancer cells exhibiting stemness features. EMT induction and CSC emergence are considered major causes of chemoresistance^2,3,11–15^ and could therefore explain the chemoresistance linked to CoPEC infection. The co-emergence of EMT and CSCs in response to CoPEC accorded with the revised view of CSC and EMT, in which EMT is a way to access stemness.^2,7,9,10,16,71^ In the stomach, the carcinogen *Helicobacter pylori* drives EMT.^72,73^ Epithelial feature alteration by EMT might therefore be a mechanism shared by pathogenic microbial communities involved in cancer progression.

Compared to other CSC markers (OCT-3/4, NANOG, etc.), the percentage of cells positive for CD133 upon 11G5 infection might appear low. However, it should be noted that CD133 regulation is complex; it involves several transcription factors (OCT4, SOX2, HIF, NICD) and therefore the physiological context of cells.^74^ Furthermore, even if OCT4 and SOX2 are overexpressed and are localized in the nucleus upon CoPEC infection (Figure 4a), we did not identify on which promoters they are bound. It should be interesting to perform a ChIP-seq analysis of 11G5-infected cells in order to decipher on which promoters OCT4, SOX2 … bind.

This role of CoPEC in the promotion of EMT and CSCs was supported by the induction of EMT and CSC markers by the secretory phenotype of colon cancer cells made senescent by CoPEC infection. Cellular senescence – a state of cell cycle arrest usually considered to be stable – is a key mechanism for countering tumorigenesis by halting the proliferation of damaged cells.^75^ However, senescent cells secrete a complex mix of cytokines, chemokines, matrix metalloproteinases (MMP), and growth factors, known as SASP.^75,76^ Senescence induction and SASP stigmas have been observed in response to CoPEC infection *in vitro*, in CRC mouse models, and in human CRC biopsies.^38,39^ CoPEC-associated SASP taken from colon cancer cells comprised IL-6, MMP-3, and growth factors, including fibroblast growth factor (FGF) and hepatocyte growth factor (HGF),^38,77^ which are inducers of EMTs.^78^ By inducing senescence and the corresponding secretory phenotype, CoPEC not only exerts selection pressure that enhances the expansion of senescence-resistant CSCs but indirectly promotes a tumor microenvironment favorable to CSC emergence *via* the EMT pathway in agreement with the key role of the tumor microenvironment in the regulation of CSC compartments.^71,79,80^

In conclusion, our data supported the hypothesis that CoPEC can play a role in CRC progression and resistance to chemotherapy. Previous studies have shown that CoPEC can induce DNA damage, leading to mutations and chromosomal instability, which may be involved in cancer initiation. Our results showed that they can also affect the fate of tumors through the promotion of EMT and CSCs, which can affect cancer progression and susceptibility to treatments including chemotherapies and the therapy anti-PD1.^81^

## Materials and methods

### Bacterial strains

The clinical 11G5 CoPEC strain, its isogenic mutant depleted for the *clbQ* gene in the *pks* island (11G5*Δpks*) unable to produce colibactin,^38^ and the transcomplemented 11G5*Δpks* strain (11G5*Δpks:pks*),^38^ were grown at 37°C in Luria-Bertani (LB) medium overnight. Bacterial inoculums were assessed at OD_620 nm_ using a NanoPhotometer® (Implen GmbH, Munich, Germany).

### Cell culture

The human intestinal epithelial cells HT-29 (ATCC, HTB38) were maintained in an atmosphere containing 5% CO_2_ at 37°C in the culture medium recommended by ATCC (American Type Culture Collection).

### Infection and preparation of conditioned medium (CM)

The cells were infected as previously described.^38^ Briefly, after 3 h of infection at a multiplicity of infection (MOI) of 500, cells were extensively washed with PBS and a culture medium containing 200 μg/mL of gentamicin was added. The culture medium was changed every 2 days. For the preparation of a conditioned medium, 5 days post-infection, cells were washed with PBS and a culture medium without serum was added. Sixteen hours later, the medium was collected (as a conditioned medium) and used to culture uninfected cells for the indicated time. The conditioned medium was changed every 2 days.

### Culture in soft agar

Three weeks post-infection, HT-29 cells were trypsinized, seeded in a culture medium with 0.7% soft agar, and plated on 12-well plates (2,500 cells/well). The culture medium was changed every 2 days (1 mL/well). After 2 weeks, cells were stained using 0.05% crystal violet.

### Culture using ultra-low attachment plates

Three weeks post-infection, the cells were trypsinized and 200,000 cells were plated in 6-well plates (ultra-low attachment plates; Corning® Inc., NY, USA). After 7 days, the cells were washed with PBS, stained with Giemsa stain, and counted.

### Alkaline phosphatase activity

Three weeks post-infection, the cells were washed with PBS and fixed with 3.7% paraformaldehyde for 3 min at room temperature. After several washes with PBS, cells were incubated for 2 h at room temperature in darkness with the alkaline phosphatase substrate BCIP®/NBT, according to the manufacturer’s instructions (SIGMAFAST™ BCIP®/NBT; Sigma-Aldrich, MO, USA).

### Protein extraction and western blot analysis

Cell and tissue samples were lysed in radioimmune precipitation assay buffer (150 mM NaCl, 0.5% sodium deoxycholate, 50 mM Tris-HCl, pH 8, 0.1% SDS, 0.1% Nonidet™ P -40) supplemented with protease inhibitors (Roche AG, Switzerland). Proteins were separated on SDS/PAGE gels, transferred to nitrocellulose membranes, and blocked with 5% nonfat milk in PBS containing 0.1% Tween® 20. The membranes were then probed overnight at 4°C with the relevant primary antibodies: anti-E-cadherin (Cell Signaling Technology, MA, USA), anti-vimentin (Cell Signaling Technology), anti-SNAIL (Thermo Fisher Scientific, MA, USA), anti-ZEB1 (Cell Signaling Technology), anti-OCT3/4 (Cell Signaling Technology), anti-NANOG (Cell Signaling Technology), and anti-GAPDH (Cell Signaling Technology). After extensive washing, the membranes were incubated with the appropriate HRP-conjugated secondary antibodies (Cell Signaling Technology). Blots were detected using an enhanced chemiluminescence detection kit (Amersham BioSciences, UK) and revealed using the ChemiDoc^TM^ XRS System (Bio-Rad Laboratories, CA, USA).

### Fluorescent microscopy

Three weeks post-infection, cells were trypsinized, seeded on coverslips, fixed with 4% paraformaldehyde, permeabilized with 0.5% Triton™ X-100 for 20 min, and blocked for 1 h with PBS containing 0.025% Triton™ X-100, 3% BSA, and 5% FBS. Cells were immunostained overnight at 4°C with the indicated primary antibodies: anti-c-MYC (Cell Signaling Technology), anti-SOX2 (Cell Signaling Technology), anti-NANOG (Cell Signaling Technology), anti-KLF4 (Abcam, UK), anti-OCT3/4 (Cell Signaling Technology), anti-N-cadherin (Cell Signaling Technology), and anti-vimentin (Cell Signaling Technology). After washing with PBS, slides were incubated with appropriate secondary antibodies coupled with Alexa488™ or Cy3 dyes (Molecular Probes, OR, USA). Nuclei were stained with DAPI (Sigma-Aldrich, MO, USA). Coverslips were then mounted with Mowiol® solution (Calbiochem®; Sigma-Aldrich, MO, USA), and the slides were examined with a Zeiss LSM 510 Meta (ZEISS, Germany) confocal microscope. Each microscopy image represents three independent experiments.

### Quantitative real-time RT-PCR (qRT-PCR)

Total RNAs were isolated using TRIzol® reagent (Thermo Fisher Scientific) following the manufacturer’s instructions. Two micrograms of mRNA were reverse transcribed using a first-strand cDNA synthesis kit (EUROMEDEX, France), and qRT-PCRs were performed using MESA BLUE qPCR kits for SYBR® assay (Eurogentec, Belgium) on a Mastercycler Realplex^4^ (Eppendorf, Germany) with specific primers (see Supplemental Table S1). 36B4 was used as an internal control for the quantification of mRNA expression. Fold induction was calculated using the *Ct* method, and the final data were derived from 2^−ΔΔ*Ct*^.

### Quantification of CD133 positive cells using flow cytometry

Briefly, 3 weeks post-infection, cells were trypsinized and 10^6^ cells were resuspended in 45 µl of PBS containing 2 mM of EDTA and 2% FBS. After the addition of 20 µl of FCR blocking reagent (Miltenyi Biotec, Germany), the cells were incubated at 4°C for 10 min with anti-CD133 antibody (Miltenyi Biotec) following the manufacturer’s instructions. Before the analyses, the cells were washed twice with PBS containing 2 mM EDTA and 2% FBS, and 7-AAD (0.25 µg/reaction; BD Biosciences, CA, USA) was added to detect dead cells. Cell populations were detected using a BD FACSAria™ SORP (BD Biosciences). For gating, we only considered singlets viable cells.

### In vitro drug resistance assays

Three weeks post-infection, the cells were trypsinized, seeded in 96-well plates (5 × 10^3^/well), and cultured for 1 week with chemotherapeutic drugs. In order to assess the impact of the conditioned medium on HT-29 cells’ resistance to chemotherapeutic drugs, HT-29 cells were seeded in 96-well plates (5 × 10^3^/well) and cultured for 1 week with CM derived from 11G5-infected HT-29 cells or from 11G5*Δpks*-infected HT-29 cells supplemented with chemotherapeutic drugs. Cell viability was assessed using the 3-(4,5-dimethylthiazol-2-yl)-2,5-diphenyltetrazolium bromide (MTT) assay.

### AOM/DSS mouse model

Six-to-eight-week-old C57BL/6 mice (Charles River Laboratories, Ecully, France) were intraperitoneally injected with AOM (10 mg/kg body weight; Sigma-Aldrich, MO, USA) and treated with streptomycin in drinking water (2 mg/mL) for 2 days to facilitate CoPEC colonization.^38^ Mice were allowed to drink regular water for 24 h and received 10^9^ colony forming units of 11G5 or 11G5*Δpks* by gavage. Five days later, their drinking water was supplemented with 2% dextran sodium sulfate (DSS, colitis grade (36,000–50,000); MP Biomedicals, Illkirch-Graffenstaden, France) for 1 week. The mice then received regular water for 2 weeks. This cycle (1 week of DSS; 2 weeks of regular water) was repeated once, and the mice were then sacrificed. Colon tumors were collected using a dissecting microscope, immediately frozen in liquid nitrogen, and stored at −80°C until protein extraction. None of the mice were removed.

### Xenograft mouse model

Three weeks post-infection, HT-29 cells were trypsinized; then, 10^6^ cells were embedded in a growth factor-reduced Matrigel®(Becton Dickinson, NJ, USA) and subcutaneously injected into the dorsal flaps of 5-week-old female nude mice (Charles River Laboratories, Ecully, France). Seven days post-engraftment, mice received 30 mg/kg of 5-FU twice weekly for 3 weeks. The tumor sizes were measured using calipers twice a week. In order to assess the impact of the conditioned medium on HT-29 cells’ resistance to chemotherapeutic drugs *in vivo*, HT-29 cells were cultured for 5 days with a conditioned medium derived from 11G5-infected HT-29 cells or 11G5*Δpks*-infected HT-29 cells. The cells were trypsinized, then 10^6^ cells were embedded in growth factor-reduced Matrigel® and subcutaneously injected into the dorsal flaps of 5-week-old female nude mice. Seven days post-engraftment, the mice received 30 mg/kg of irinotecan twice weekly for 3 weeks. Tumor sizes were evaluated twice weekly with caliper measurements using the following formula: tumor volume = (length × width^2^/2). Relative tumor growth inhibition was calculated by the relative tumor growth of the treated mice divided by the relative tumor growth of the control mice following the initiation of therapy. None of the mice were removed.

### Ethics statement

The animal protocols were approved by the French Ministry of Education, Research and Innovation (APAFIS permits numbers #4096, #4099), and all animals were used in accordance with the European Community guidelines for the care and use of animals (86/609/CEE).

Samples from CRC patients were collected, according to previously published studies,^34,38^ from colon resections that were required for the treatment of the patients. Ethical approval for the study was granted by the Clermont-Ferrand Research Ethics Committee. Verbal informed consent to participate in the research was obtained from all the patients included in the study in accordance with French bioethics law (Act No. 2004-800 of August 6, 2004). Samples were taken from the resected colons at the site of malignant tumors. Pathological analysis confirmed the neoplastic features of the samples.

### Statistical analysis

Values are expressed as means ± SEM. Statistical analyses were performed with GraphPad Prism version 5.01 software using a two-tailed Student’s t-test or a Mann-Whitney U-test, depending on the results of a D’Agostino-Pearson omnibus normality test. When appropriate, a one-way ANOVA test with a Bonferroni post hoc test was performed.

## Supplementary Material

Supplemental Figures revised version.docx

supplementary_data.docx

## Data Availability

The data that support the findings of this study are available from the corresponding author, GD, upon reasonable request.
