# Supplementary material for: Colibactin-producing Escherichia coli enhance resistance to chemotherapeutic drugs by promoting epithelial to mesenchymal transition and cancer stem cell emergence
Source: Gut Microbes. 2024 Feb 19;16(1):2310215. doi: 10.1080/19490976.2024.2310215 (PMC10880512; doi:10.1080/19490976.2024.2310215)
Supplement: Supplemental Figures revised version.docx [file KGMI_A_2310215_SM0335.docx]

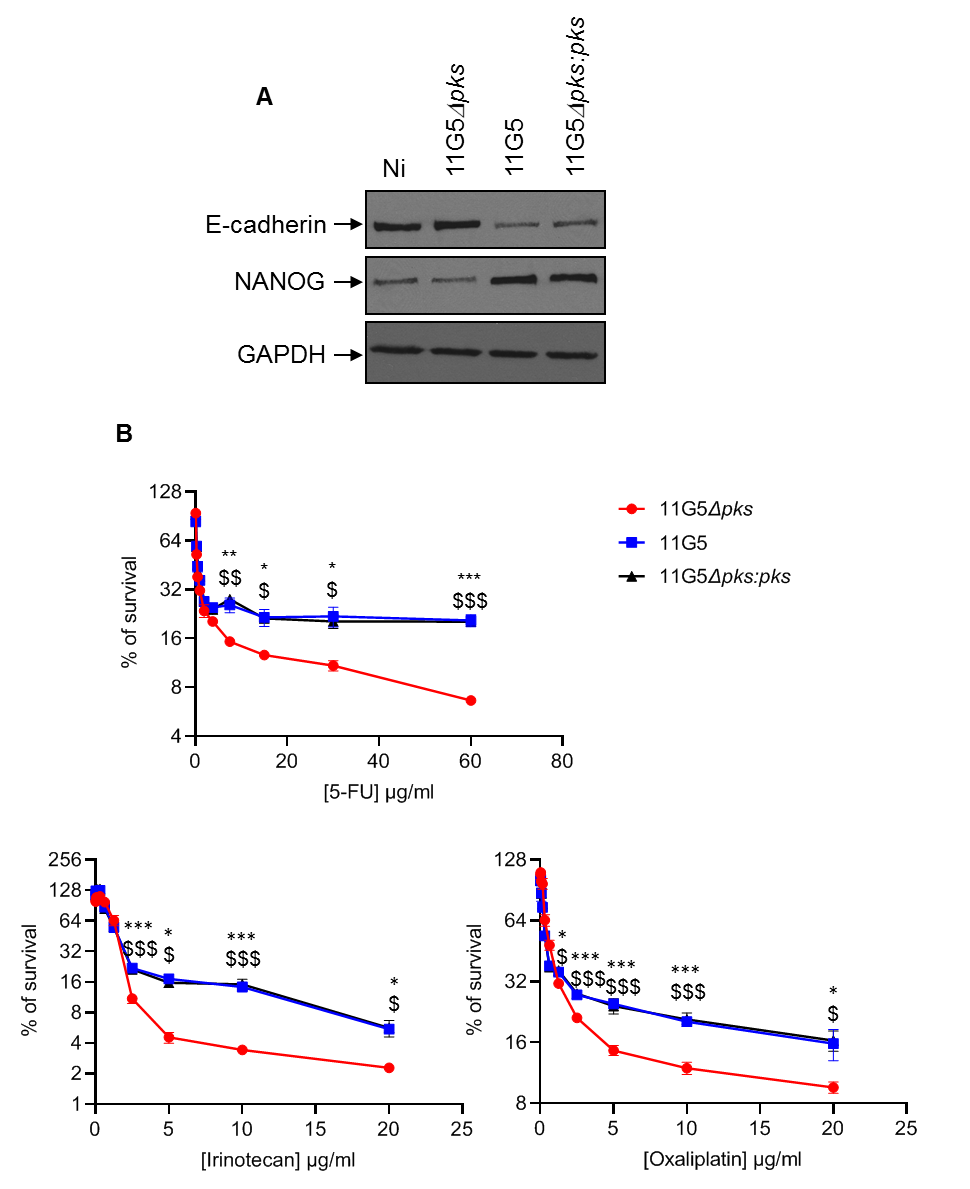


**Supplemental Figure 1. Transcomplentation of *clbQ* restores the wild-type phenotype of the 11G5*Δpks* strain.** HT-29 cells were uninfected (Ni), infected with the wild-type 11G5 strain, the 11G5*Δpks* strain or the transcomplemented 11G5*Δpks* strain (11G511G5*Δpks:pks*), and 3-week post-infection cells were used. (**A**) E-cadherin and NANOG were analyzed using Western blot. (**B**) Cells were trypsinized, seeded on 96-well plates, and exposed to various doses of chemotherapeutic drugs for 1 week. Cellular viability was assessed by MTT assay. Uninfected cells were used to represent 100% viability. Data are means ± SEM of eight replicates and are representative of three independent experiments. **P* < 0.05; ***P* < 0.005; ****P* < 0.001 (11G5 *vs* 11G5*Δpks*); $*P* < 0.05; $$*P* < 0.005; $$$*P* < 0.001 (11G5*Δpks:pks vs* 11G5*Δpks*).


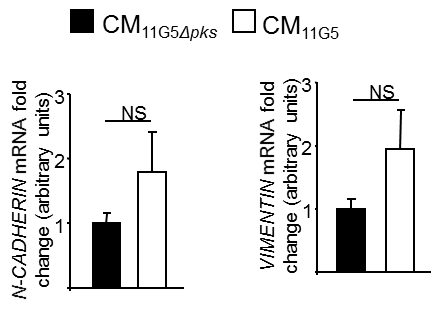


**Supplemental Figure 2.** **The SASP induced by 11G5 infection promoted the expression EMT markers.** Cells were infected with the 11G5 strain or the 11G5*Δpks* strain. 5 days post-infection, conditioned media (CM) derived from infected cells were collected and used to culture uninfected cells for 3 days: CM_11G5_, CM derived from 11G5-infected cells; CM_11G5_*_Δpks_*, CM derived from 11G5*Δpks*-infected cells. *N-cadherin* and *vimentin* mRNA levels were quantified using qRT-PCR. Values represent means ± SEM. NS, not significant.


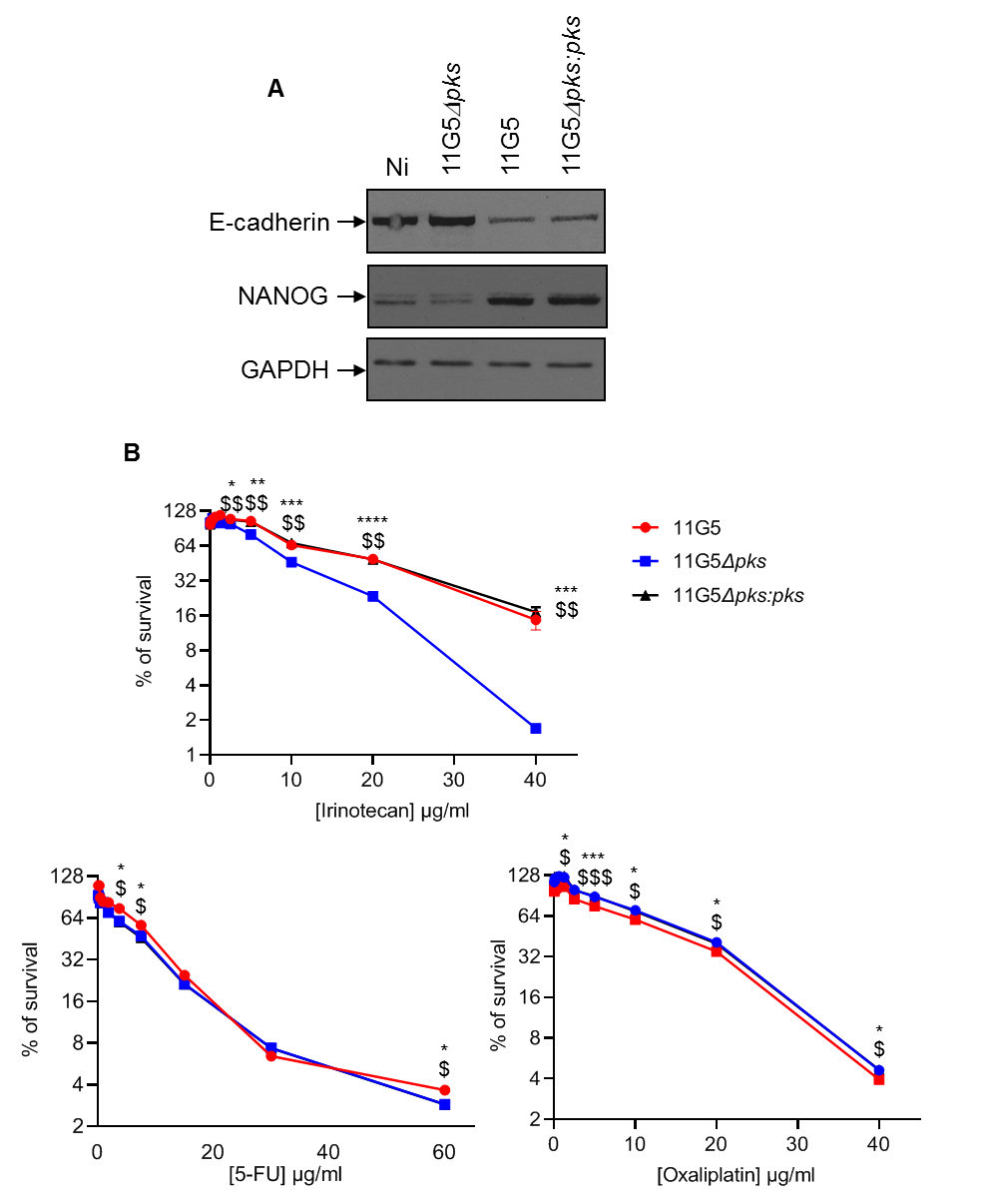


**Supplemental Figure 3. Conditioned media (CM) derived from HT-29 cells infected with 11G5*Δpks:pks* exhibited the same effect than CM derived from 11G5-infected HT-29 cells.** HT-29 cells were uninfected (Ni), infected with the wild-type 11G5 strain, the 11G5*Δpks* strain or the transcomplemented 11G5*Δpks* strain (11G511G5*Δpks:pks*). 5 days post-infection, conditioned media (CM) derived from cells were collected and used to culture uninfected cells for 5 days (**A**) or for 1 week (**B**). (**A**) E-cadherin and NANOG were analyzed using Western blot. (**B**) Cells were trypsinized, seeded on 96-well plates, and exposed to various doses of chemotherapeutic drugs for 1 week. Cellular viability was assessed by MTT assay. Uninfected cells were used to represent 100% viability. Data are means ± SEM of eight replicates and are representative of three independent experiments. **P* < 0.05; ***P* < 0.005; ****P* < 0.001; *****P* < 0.0001 (11G5 *vs* 11G5*Δpks*); $*P* < 0.05; $$*P* < 0.005; $$$*P* < 0.001 (11G5*Δpks:pks vs* 11G5*Δpks*).
