# Supplementary material for: Colibactin-producing Escherichia coli enhance resistance to chemotherapeutic drugs by promoting epithelial to mesenchymal transition and cancer stem cell emergence
Source: Gut Microbes. 2024 Feb 19;16(1):2310215. doi: 10.1080/19490976.2024.2310215 (PMC10880512; doi:10.1080/19490976.2024.2310215)
Supplement: supplementary_data.docx [file KGMI_A_2310215_SM0334.docx]

**Supplementary Table 1: Primers used in this study.**

| Primer | Sequence (5’-3’) |
| --- | --- |
| 36B4_For_ | CCAGGCTTTGGGCATCA |
| 36B4_Rev_ | CTTTATCAGCTGCACATCACTCAG |
| Oct3/4_For_ | AGGAGAAGCTGGAGCAAAAC |
| Oct3/4 _Rev_ | GCCTGTGTATATCCCAGGGTG |
| Nanog_For_ | GAGATGCCTCACACGGAGAC |
| Nanog _Rev_ | GGGTTGTTTGCCTTTGGGAC |
| E-Cadherin_For_ | TGCCCAGAAAATGAAAAAGG |
| E-cadherin _Rev_ | GTGTATGTGGCAATGCGTTC |
| N-Cadherin_For_ | TGACTATGAAGGCAGTGGCTC |
| N-Cadherin _Rev_ | CAGTCATCACCTCCACCATACAT |
| Fibronectin_For_ | TCCCTCGGAACATCAGAAAC |
| Fibronectin _Rev_ | CAGTGGGAGACCTCGAGAAG |
| Vimentin_For_ | GAGAACTTTGCCGTTGAAGC |
| Vimentin _Rev_ | GCTTCCTGTAGGTGGCAATC |
| Snail_For_ | AAGATGCACATCCGAAGCC |
| Snail _Rev_ | CGCAGGTTGGAGCGGTCAGC |
| Zeb_For_ | CCTGAGCTTCAGGCCCCAGGC |
| Zeb _Rev_ | GGTGTACCAGAGGATGACCTGCCA |

For, forward; Rev, reverse.
